# Continuous external compression for the treatment of humeral pseudarthrosis: a single center experience

**DOI:** 10.11604/pamj.2020.35.105.21533

**Published:** 2020-04-08

**Authors:** Sami Sallemi, Nizar Sahnoun, Mahdi Maatoug, Moez Trigui, Imen Zouch, Mariem Keskes, Ameur Abid, Hassib Keskes

**Affiliations:** 1 Department of Orthopedic Surgery and Traumatology, Habib Bourguiba University Hospital, Sfax, Tunisia; 2 Department of Anesthesia, Habib Bourguiba University Hospital, Sfax, Tunisia

**Keywords:** Pseudarthrosis, humerus, continuous, compression, orthofix

## Abstract

Humeral pseudarthrosis are common with a non-union rate after fracture between 8% and 13%. Several operative methods have been described for the treatment of humeral pseudarthrosis. The aim of this study was to assess a new approach based on compression using a monoplane external fixator without graft. This study was conducted in the Department of Orthopedic Surgery, and Traumatology of Habib Bourguiba University Hospital in Sfax-Tunisia between April 2009 and September 2018. Fifty-eight patients were operated on using a dynamic monoplane axial fixation device with continuous compression by manipulating the compression system of the fixator. All the cases were evaluated according to the modified Stewart and Hundley classification. The fracture was located in the middle third in 53.4% of the cases. The pseudarthrosis was hypertrophic in 34.5% of the cases. Fifty-four patients were treated with this method as a first cure of non-union and four patients had previously a first cure for their pseudarthrosis. We noted 11 septic pseudarthrosis. The average follow-up was 47.2 months. We obtained consolidation in 98% of the cases. The average consolidation time was 5.1 months. Based on the modified Stewart and Hundley criteria, 75.8% had very good results. This study highlights that a continuous external compression is effective in the treatment of non-unions, as it allows consolidation without opening the pseudarthrosis site and without bone grafting while having satisfactory anatomical and functional results.

## Introduction

Humeral shaft fractures represent 1% of skeletal fractures, with a high rate of bone healing. However, unfavorable evolutions are not exceptional, and humeral pseudarthrosis ranges from 8% to 13% [1, 2]. Several operative methods were described in the literature for treatment of humeral shaft pseudarthrosis. The dynamic external fixation with bone graft is classically used [3]. The objective of this paper was to assess the evolution of patients with humeral pseudarthrosis who underwent external continuous compression without bone grafting.

## Methods

This study was conducted in the orthopedic surgery department in Habib Bourguiba hospital in Sfax, Tunisia. It involved 58 cases with humeral pseudarthrosis, treated between April 2009 and September 2018 with a minimum follow up of 12 months. All the patients were operated on with the same surgeon, and using the same technique of dynamic monoplane axial fixation device in continuous compression by manipulating the compression system of the fixator. The location of the non-union concerned the humeral diaphysis under the lower edge of the large pectoral insertion and above the epicondylar crest. Fracture type was evaluated according to the AO classification. We evaluated our results according to the modified Stewart and Hundley classification [4] (Table 1).

**Table 1:** The modified Stewart and Hundley classification

	Pain	Limitation of elbow or shoulder mobility	Angulation
Excellent	None		Good alignment
Good	Occasional	<20°	<10°
Fair	After effort	20°-40°	>10°
Poor	Permanent	>40°	Non-union

## Results

**Figure 1:**
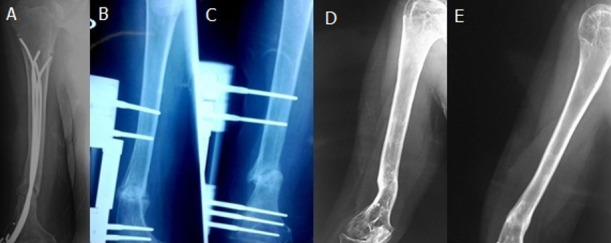
Example of a case of consolidation of humeral pseudarthrosis

**Figure 2:**
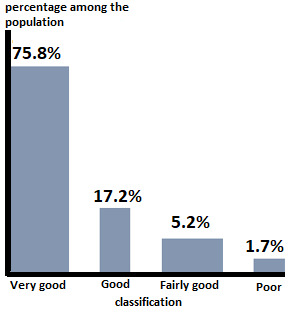
Evaluation of the patients according to the modified Stewart and Hundley

The average age of the patients was 42 years with extremes ranging from 17 to 77 years. The distribution by sex showed a clear predominance of men that represented 41 cases (70.7%) with a ratio of 2.4. The circumstances of occurrence were dominated by traffic accidents which caused 46.6% of cases. The fracture was located in the diaphyseal middle third in 53.4% of the cases and in the distal third in 46.6%. The fracture type was a simple type A in 75.9% of the cases, type B in 19%, and complex type C in 5.1%. In simple fractures, the line was transverse (A3) in 55.2% of the cases. We found 34.5% hypertrophic, 43.1% atrophic and 22.4% oligotrophic non-unions. Initially, the fracture underwent orthopedic treatment in 15.5% of the cases, an intramedullary bundle nailing in 53.4%, by screwed plate in 19% and by Hoffmann type external fixator in 12.1%. Fifty-four patients were treated with continuous external compression as a first cure of non-union and four patients had previously a first cure for their pseudarthrosis and were treated with this method after the failure of the first cure. Good alignment and bone contact were checked with intraoperative radioscopy. The average recovery period was 47.2 months, with extremes ranging from 12 to 102 months. We obtained a consolidation for 98% of the pseudarthrosis (Figure 1). Only one case did not consolidate, including a sepsis on the pins. The average consolidation time was 5.1 months with extremes ranging from 2 to 10 months (Table 2). We noted 11 sepsis on pins, 10 of which evolved well after antibiotic treatment. There were no neurological complications, or algodystrophy. Based on the modified Stewart and Hundley criteria, we found 75.8% of very good results, 17.2% of good results, 5.2% of fairly good results, and 1.8% of poor result (Figure 2).

**Table 2:** Consolidation time (months)

Consolidation time (months)		Average
Type of non-union	Atrophic	6.6
	Oligotrophic	4.3
	Hypertrophic	3.5

## Discussion

Compression is the principle adopted in our surgical procedure for the non-union of long bones. Using a compression plate is the only technique allowing a good stabilization of the non-union, while performing a bone graft is essential for consolidation. However, in this type of treatment, bone graft seems to play a crucial role. Healy *et al*. [5] reported 45% of failures by screwed plates without bone graft. Moreover, several articles reported that consolidations could only be obtained by grafting without any modification of the osteosynthesis. Interestingly, the isolated graft supply partly explains the excellent results on non-union cures by plate, bone graft sometimes being considered as an “adjuvant” to the treatment already carried out [6]. Also, we obtained a 98% consolidation rate with the use of screwed plate associated with spongy graft as shown in Table 3 [3, 5-11]. Centromedullary nail fixation preserves periosteal vascularity and decreases the rate of radial paralysis and infection. However, this technique is controversial due to the high rate of failure according to several studies as displayed in Table 4 [8, 12-15].

**Table 3:** Evaluation of the patients according to the modified Stewart and Hundley criteria

References	N	Failure	Period (months)	Infection	Radial Paralysis
Singh AK [3]	20	0	4 months	3	2 Resolved
Healy WL [5]	26	8%	5.5 months	0	1 Definitive
Chantelot C [6]	17	6%	5 months	1	0
De Dompsure RB [7]	21	15%	4.8 months	2	2 Resolved
Loomer R [8]	20	10%	3 months	1	2 Resolved
Muller ME [9]	12	0%	4 months	0	0
McKee MD [10]	9	0%	4 months	0	0
Wu CC [11]	19	10%	4.5 months	0	1

**Table 4:** Results of the treatment of non-union with nail

References	Type of nail	N	Failure	Average period
Loomer [8]	Multiple	6	0%	4 months
Pietu [14]	Seidel	5	0%	3.5 months
McKee [13]	Seidel/Russel Taylor	10	60%	5 months
Dujardin [12]	Seidel/Russel Taylor	13	40%	5 months
Wu [15]	Seidel+graft	16	13%	4.5 months

Several hypotheses could explain these failures, like the insufficiency of primary stability or the impairment of the endomedullary vascularization of the humerus [14]. The Ilizarov external fixator should allow stable fixation, possible correction of the reduction and compression of the pseudarthrosis site [16, 17]. Table 5 shows the good consolidation rate of the Ilizarov device [17-19]. Ilizarov external fixator preserves the periosteum vascularization. However, this method is associated with a significant rate of complications including stiffness, iterative fractures after removal of the fixator, nerve damage (axillary nerve on the proximal pins or of the radial nerve on the distal pins) and the very bulky material. The consolidation rate is around 90% in 6 months with 10% disabling stiffness in the shoulder or elbow. We adopted the principle of compression by external fixation inspired by Ilizarov data on the confrontation of the edges of the non-union, crushing of the interposing tissues, creation of micro fractures, local necrosis of the interposed fibro-cartilaginous tissues, inflammation and thus an influx of polynuclear cells, and osteoclasts followed by osteoblasts, thus initiating osteogenesis [20, 21]. Monoplane external fixator in compression allowed a minimal unilateral exposure at the non-union site, minimal surgical trauma to the soft tissue, an easy application of the fixator and consecutively minimal blood loss and a short operating time [22]. This technique was first described in 2001 by Lavini F *et al*. [23] about 27 aseptic pseudarthrosis of the humerus but associated in 26 cases with bone graft. Only one case was treated by simple compression without opening the site. The consolidation rate was 92.5% within a period of 4.9 months. No major complications were observed (radial paralysis, joint stiffness, or deep infection). For Atalar AC *et al*. [24], 24 patients were treated with an external monoplane fixator in compression, with bone graft for all patients. The consolidation rate was 95.8%, within a period of 5.2 months. In our series, 58 patients were treated with an external monoplane compression fixator. No cortico-spongy graft was made. The opening of the site was only necessary in 43.1% of the cases. The consolidation rate was 98%, within an average time of 5.1 months.

**Table 5:** Results of the treatment of non-union with an Ilizarov type external fixator

References	Type of fixator	Number of cases	Rate of consolidation	Period
Raschke M [17]	Ilizarov	1	100%	4 months
Patel [18]	Ilizarov	6	83%	6 months
Lammens [19]	Ilizarov	24	96%	4.5 months

Compared to the literature, the treatment of humeral non-union by monoplane external fixation with compression reduces the operating time, minimizes bleeding and avoids bone grafting and vascular-nerve complications. It also reduces the duration of hospitalization while obtaining very satisfactory anatomical and functional results, whatever the type of non-union. The study shows that the use of an external compression fixation in the treatment of humeral pseudarthrosis allows their consolidation without opening the pseudarthrosis site and without bone graft. Compression was shown to allow the necrosis of the interposition tissues and the bone contact associated with a stable fixation. These conditions could be maintained throughout the treatment by the possibility of secondary compression during follow-up. The opening of the pseudathrosis site is only necessary for removal of material or for radial nerve neurolysis. The only limit to this technique is sepsis on pins.

## Conclusion

The treatment of humeral pseudarthrosis with monoplane external fixator with compression seems effective regarding the low incidence of complications and the high rate of bone healing. This method is a good indication in the treatment of humeral pseudarthrosis and probably long bone pseudarthrosis in general.

### What is known about this topic

- Humeral pseudarthrosis are not uncommon;
- Their treatment is not unanimous;
- Many surgical procedures are proposed including compression by plate associated with bone graft.

### What this study adds

- External continuous compression is an efficient and reliable method;
- External continuous compression is easy to do and at low complication rate;
- External continuous compression gives excellent functional results.

## Competing interests

The authors declare no competing interests.
